# Assessment of the efficiency and accuracy of an artificial intelligence assistive system in the diagnosis of Pap cervical atypical glandular cell cytology

**DOI:** 10.1002/cncy.70056

**Published:** 2025-10-09

**Authors:** Xin Zhang, Xing Dong, David Starr, Xinru Bai, Yingbin Li, Terri E. Jones, Yixuan Liu, Xiaoqian Wang, Changzhong Li, Xiangchen Wu, Xianxu Zeng, Chengquan Zhao

**Affiliations:** ^1^ Department of Pathology The Third Affiliated Hospital of Zhengzhou University Zhengzhou China; ^2^ Department of Pathology University of Pittsburgh Medical Center Pittsburgh Pennsylvania USA; ^3^ Ruiqian Technologies Company, Ltd Suzhou China

**Keywords:** AICyte, artificial intelligence, atypical glandular cells (AGCs), cervical cancer, Papanicolaou cytology

## Abstract

**Background:**

A diagnosis of atypical glandular cells (AGC) on Papanicolaou (Pap) slides is rare but has clinically significant findings associated with high‐risk cervical and endometrial lesions. The authors evaluated the efficiency and diagnostic performance of an artificial intelligence (AI)‐assisted platform (Riuqian WSI‐2400; with the registered trademark AICyte) in identifying AGCs on Pap slides.

**Methods:**

A retrospective analysis of 485 Pap cases was conducted, including 185 cases with AGCs, 50 cases with high‐grade squamous intraepithelial lesions, 50 cases with low‐grade squamous intraepithelial lesions, and 200 negative cases; of these, 264 cases had histologic correlations. An experienced cytopathologist reviewed all slides using conventional microscopy and AICyte. Then, the same cases were evaluated by two other pathologists using the AICyte system.

**Results:**

The initial study demonstrated a kappa value of 0.744, which indicated strong agreement of the Pap interpretation from the same pathologist between using microscopy and AICyte methods, whereas the average interpretation time was significantly reduced with AICyte (137 vs. 44 seconds). Diagnostic consensus among three pathologists using the AICyte system was strong, with a Kendall W coefficient of 0.802. The AICyte‐pathologist consensus reached an exact match with original interpretations in 95.1% of cases. AICyte‐assisted interpretations demonstrated improved specificity and diagnostic accuracy for glandular lesions compared with original interpretations while maintaining 100% sensitivity and negative predictive value.

**Conclusions:**

To the authors' knowledge, this is the first study focusing on assessment of AGCs on an artificial intelligence system. The findings demonstrated that the AICyte system offers substantial improvements in efficiency and diagnostic consistency for the interpretation of AGCs and significantly reduces slide reading time. These results support the potential of AI to augment performance, especially in resource‐limited settings or high‐volume screening environments.

## Abstract 2

The efficiency and diagnostic performance of an artificial intelligence‐assisted system for assessing Papanicolaou slides were investigated and appeared to shorten the time to diagnosis compared with manual microscopy. The results demonstrated the potential utility and applicability of the system in cervical cytopathology for assisting pathologists in recognizing atypical glandular cells and equivalent diagnoses in a more efficient manner.

## INTRODUCTION

The diagnosis of atypical glandular cells (AGCs) is used to acknowledge the presence of glandular cells exhibiting cytologic atypia that is more pronounced than inflammatory or reactive change but is not sufficiently aberrant to be classified as malignant in Papanicolaou (Pap) cytology slides.^1^ AGCs are considered rare on Papanicolaou (Pap) tests, representing approximately 0.15%–1.00% of cases.^1^, ^2^, ^3^, ^4^ Although uncommon, AGCs represent a high‐risk cytology category, with the potential of identifying precancerous and cancerous lesions of the cervix and/or endometrium on histologic follow‐up in up to 35% of cases.^1^, ^4^, ^5^, ^6^ Although the accurate diagnosis of AGCs is important, the diagnosis of AGCs remains challenging, primarily because of morphologic overlap with reactive and squamous lesions. This diagnostic conundrum is reflected in poor concordance rates even among experienced pathologists.^7^, ^8^, ^9^ Another challenge is human papillomavirus (HPV) co‐testing results: whereas a positive HPV status can be helpful in detecting assistance for lesions of cervical origin, endometrially derived lesions, which are not associated with HPV, would rely solely on cytomorphology for diagnosis.^10^, ^11^, ^12^ In addition, the rate of HPV‐negative endocervical cancers, especially the endocervical gastric type, can be relatively higher in some areas and countries.^11^, ^12^ These issues raise the question of whether future technology will have a role to play in increasing pathology diagnostic accuracy, with artificial intelligence (AI) as the main source of innovation.

The role of AI in gynecologic cytology continues to be expanded and enriched. AI has been applied to the diagnosis of squamous lesions in many studies,^13^, ^14^, ^15^, ^16^, ^17^, ^18^, ^19^ and future work into assessing its role in diagnosing AGCs becomes necessary. Challenges in training AI models for AGC detection stem from the lower incidence of AGCs compared with squamous lesions on Pap slides, an overlap in morphology with high‐grade squamous lesions, and discrepancies in interpretation between investigators. These factors can make training an AI model difficult if distinguishing AGCs is not the primary focus.^13^, ^14^, ^15^, ^16^, ^17^, ^18^, ^19^

The AICyte system (Ruiqian Technologies Company, Ltd.) was successfully applied to the diagnosis of squamous lesions on Pap slides in a previous study, which demonstrated that the specificity of AICyte in detecting cervical intraepithelial neoplasia (CIN) 2 or greater (CIN2+) lesions was significantly increased (16.19% vs. 6.77%) compared with the original ThinPrep interpretation (OTPI). The study also indicated that AICyte decreased the time to diagnosis of squamous lesions compared with the OTPI.^20^ In addition, the 50% negative cutoff value of the AICyte‐only interpretation demonstrated high sensitivity and negative predictive value (NPV). This would substantiate AICyte as a suitable candidate for optimizing workflow, especially for scenarios in which there are workplace shortages of cytotechnologists or available pathologists.^21^ In these previous studies, it was noted that the primary focus was on assessing squamous lesions because that is the main function of AICyte; however, a subset of cases included in the squamous studies with the AICyte system performed diagnoses of AGCs.^20^, ^21^ Therefore, the objective of the current study was to put the classification of AGCs in the forefront to determine the extent of AICyte's capability for detecting AGCs on Pap slides.

## MATERIALS AND METHODS

### AICyte development

This study was conducted using the Ruiqian WSI‐2400 (with the registered trademark AICyte), a fully automated cytology scanner. The AICyte scanner uses real‐time autofocus on liquid‐based cytology slides to demonstrate relevant cytologic images for screening purposes. AICyte uses an object‐detection method based on an AI deep‐learning convolutional neural network YOLO5 model (Ultralytics). After scanning, a whole‐slide image and relevant AI diagnostic data, presented as 20 of the most diagnostically relevant images, are made available for pathologist review.^20^, ^21^ The current AICyte cell classifier was updated by using 533,997 expert‐annotated cell images, including 72,369 images of atypical squamous cells of undetermined significance (ASC‐US); 24,390 images of atypical squamous cells, cannot exclude high‐grade squamous intraepithelial lesion (ASC‐H); 4312 images of AGCs; 81,082 images of low‐grade SIL (LSIL); 51,280 endocervical cells; 7182 images of cervical metaplastic cells; 255,597 images of negative cells; and 37,785 images of HSIL images as a cell training database. Furthermore, the AICyte specimen classifier was trained by using 30,056 expert‐diagnosed slides that were negative for intraepithelial lesion or malignancy (NILM), 4170 LSIL slides, 4109 ASC slides, 1271 HSIL slides, 367 AGC slides, and 3198 ASC‐H slides.

### Case selection

A retrospective study was conducted after approval was obtained from the Institutional Review Board of the Third Affiliated Hospital of Zhengzhou University (TAHZU). Informed consent was waived because of the retrospective nature of this study. One‐hundred eighty‐five Pap tests with an original interpretation of AGCs (88 cases of AGC‐not otherwise specified, 43 cases of AGC‐endometrial, 29 cases of AGC‐endocervical, 25 cases of AGC‐favor neoplastic) diagnosed between January 2020 and July 2024 were obtained from the pathology database at TAHZU. The average age of the patients was 45 years (age range, 21–80 years). Additional categories of Pap cytology cases were selected and admixed with AGC cases as a study cohort, including 50 cases of HSIL, 50 cases of LSIL, and 200 cases of NILM. Histologic follow‐up results within 6 months of the Pap tests were identified and recorded.

### Liquid‐based cytologic specimen processing

All cervical samples were collected by gynecologists and were prepared using ThinPrep 2000 system (Hologic, Inc.) according to the manufacturer's instructions. The Pap tests were screened, interpreted, and reported by 10 pathologists at the Department of Pathology at TAHZU using The Bethesda System for Reporting Cervical Pathology (TBS) 2014 criteria and terminology. The Pap tests were signed out by the pathologists in the TAHZU Department of Pathology, and relatively junior pathologists (working experience <10 years) screened the Pap tests and provided their interpretations. Then, cases were reviewed and signed out by relatively senior pathologists (>10 years of working experience).^20^

### Study design

The 485 selected Pap cytology slides were cleaned, and any prior dots signifying clusters of interest were removed. They were then randomly mixed and relabeled. In total, three pathologists, pathologist A (Path‐A), pathologist B (Path‐B) and pathologist C (Path‐C), were involved in this study. Two stages of the study were performed. In the first stage, Path‐A (with 20 years of cytologic sign‐out experience) reviewed ThinPrep interpretation on microscopy (RTPI) and recorded the review time and results. After Path‐A completed the review of all cases under the microscope, the slides were cleaned, mixed, and relabeled again. The 485 slides were then scanned and analyzed by the AICyte system. The second stage involved Path‐A, Path‐B (with 15 years of experience), and Path‐C (with 10 years of experience) reviewing the 20 objects of interest generated by AICyte and providing an interpretation for each case. Except for the patient's age, no other clinical information was provided during the review process. There was a 1‐month washout period between review on microscopy and on AICyte.

The final diagnosis from the AICyte‐assisted review reflected the same diagnosis rendered by at least two pathologists. For cases in which there were three different interpretations, the slides were re‐reviewed on AICyte for a consensus. To avoid confusion in the result analysis, reviewers were not required to distinguish additional subcategories of AGCs (such as AGC‐not otherwise specified, AGC‐favor neoplastic, AGC‐cervical, AGC‐endometrial cells, etc.) in this study.

### Statistical analysis

The consistency of the Pap diagnoses among the OTPI, the RTPI, and the interpretation by a pathologist on AICyte (AICyte‐assisted) for the different categories was assessed using the Kendall coefficient of concordance (W). The sensitivity, specificity, positive predictive value (PPV), and NPV for the detection of clinically significant biopsy results were calculated for a 95% confidence interval (CI).

## RESULTS

### Analysis of the time for Pap interpretation by AICyte or microscopy

The reading time was recorded by the pathologist during the review of the study cases under the microscope and on the AICyte system. Path‐A exhibited a mean reading time of 137 seconds per slide when using a microscope, whereas this time was reduced to 44 seconds per slide when using the AICyte system. Similarly, Path‐B and Path‐C achieved mean reading times of 43 and 40 seconds per slide, respectively, on the AICyte system.

### Comparison of cytologic interpretations under microscopy and on AICyte by Path‐A

Table 1 lists TBS categories among the OTPI, RTPI, and AICyte‐assisted interpretations performed by Path‐A. Of 485 cases, AGCs were diagnosed in 185 cases (38.2%) in the OTPI, 166 cases (34.2%) in the RTPI, and 184 cases (37.9%) in the AICyte‐assisted interpretation; and HSIL was reported in 50 cases (10.3%) in the OTPI, 48 cases (9.9%) in the RTPI, and 53 cases (10.9%) in the AICyte‐assisted interpretation. An analysis of the Kendall coefficient of concordance demonstrated almost perfect agreement (W = 0.845) for overall TBS categories between the three methods. The overall exact match rate was 83.1% (403 of 485 cases) between the Path‐A RTPI and the Path‐A AICyte interpretation. The concordance between the RTPI and the AICyte interpretation was strong with a kappa value of 0.744.

**TABLE 1:** Cytologic interpretation of the 485 cases from pathologist A.

TBS categories	No. (%)		
	OTPI	RTPI	AICyte
AGC	185 (38.2)	166 (34.2)	184 (37.9)
HSIL	50 (10.3)	48 (9.9)	53 (10.9)
LSIL	50 (10.3)	49 (10.1)	47 (9.7)
NILM	200 (41.2)	222 (45.8)	201 (41.5)

Abbreviations: AGC, atypical glandular cells; HSIL, high‐grade squamous intraepithelial lesion; LSIL, low‐grade squamous intraepithelial lesion; NILM, negative for intraepithelial lesion or malignant; No, number; OTPI, original ThinPrep interpretation; RTPI, review ThinPrep interpretation on microscopy; TBS, The Bethesda System for Reporting Cervical Cytology.

### Diagnostic consensus between three pathologists on the AICyte system

Table 2 summarizes TBS results for 485 Pap cases evaluated by three pathologists using the AICyte system. AGC reporting rates were 37.9%, 34.8%, and 30.3% by Path‐A, Path‐B, and Path‐C, respectively. The reporting rates of HSIL and LSIL cases were similar among three pathologists (Table 2). In the OTPI, 200 of 485 cases (41.2%) were interpreted as NILM in the study cohort. The NILM reporting rate was 49.1% by Path‐C, which was significantly higher than that reported by Path‐B (43.7%) and Path‐A (41.5%). The Kendall W coefficient, which measures the concordance in diagnostic categorization among multiple raters, was 0.802 for all cytologic categories.

**TABLE 2:** Comparison of interpretation of 485 Papanicolaou test cases on the AICyte system among three pathologists.

TBS categories	Pathologist A	Pathologist B	Pathologist C	
	No. of cases	%	No. of cases	%	No. of cases	%
AGC	184	37.9	169	34.8	147	30.3
HSIL	53	10.9	43	8.9	49	10.1
LSIL	47	9.7	61	12.6	51	10.5
NILM	201	41.5	212	43.7	238	49.1

Abbreviations: AICyte, artificial intelligence (AI) assisted platform; AGC, atypical glandular cells; HSIL, high‐grade squamous intraepithelial lesion; LSIL, low‐grade squamous intraepithelial lesion; NILM, negative for intraepithelial lesion or malignant; No, number; TBS, The Bethesda System for Reporting Cervical Cytology.

### Concordance between the OTPI and the AICyte‐pathologist interpretation

Among 485 cases, 478 (98.6%) had the same diagnoses by at least two pathologists. Three pathologists re‐reviewed the seven cases with three different interpretations to obtain final consensus diagnoses. Table 3 provides the results of the final AICyte‐pathologist interpretation versus the OTPI for the 485 Pap cases. The reporting rate of the AICyte pathologist for AGCs and HSIL was 36.5% and 9.5%, respectively, which was slightly lower than the reporting rate of the OTPI analysis (38.2% and 10.3%, respectively). The interpretation of LSIL by AICyte pathologist was similar to that in the OTPI (10.5% and 10.3%, respectively). Two hundred eleven cases (43.5%) were interpreted as NILM on AICyte pathology, which was slightly higher than the 200 NILM cases (41.2%) interpreted in the OTPI. The Kappa value of the AICyte‐pathologist and OTPI results was 0.925. Table 4 provides the exact interpretation concordance between the OTPI and the AICyte‐pathologist interpretation. The overall exact match rate was 95.1% (461 of 485 cases) between the two methods, with 169 cases of AGC, 46 cases of HSIL, 50 cases of LSIL, and 196 cases of NILM. Of the 185 cases of AGCs in the OTPI, 169 were diagnosed as AGCs, 15 were diagnosed as NILM, and one was diagnosed as LSIL by in the AICyte‐pathologist interpretation. Of 50 cases of HSIL cases in OTPI, 46 cases were diagnosed as HSIL, and four cases were diagnosed as AGCs on the AICyte‐pathologist interpretation. All 50 cases of LSIL in the OTPI were diagnosed as LSIL in the AICyte‐pathologist interpretation, indicating the highest concordance.

**TABLE 3:** Comparison of The Bethesda System reporting rates between the original ThinPrep and AICyte‐pathologist interpretations for 485 Papanicolaou tests.

TBS categories	AICyte pathologist			OTPI		
	No. of cases	%	95% CI	No. of cases	%	95% CI
AGC	177	36.5	32.20–40.95	185	38.2	33.80–42.63
HSIL	46	9.5	7.03–12.45	50	10.3	7.75–13.37
LSIL	51	10.5	7.93–13.59	50	10.3	7.75–13.37
NILM	211	43.5	39.04–48.05	200	41.2	36.82–45.76

Abbreviations: AGC, atypical glandular cells; CI, confidential interval; HSIL, high‐grade squamous intraepithelial lesion; LSIL, low‐grade squamous intraepithelial lesion; NILM, negative for intraepithelial lesion or malignant; No, number; OTPI, original ThinPrep interpretation; TBS, The Bethesda System for Reporting Cervical Cytology.

**TABLE 4:** Exact interpretation concordance between original ThinPrep and AICyte‐pathologist interpretations.

AICyte pathologist	OTPI, No.				
	AGCs	HSIL	LSIL	NILM	Total
AGC	169	4	0	4	177
HSIL	0	46	0	0	46
LSIL	1	0	50	0	51
NILM	15	0	0	196	211
Total	185	50	50	200	485

Abbreviations: AGC, atypical glandular cells; HSIL, high‐grade squamous intraepithelial lesion; LSIL, low‐grade squamous intraepithelial lesion; NILM, negative for intraepithelial lesion or malignant; No, number; OTPI, original ThinPrep interpretation; TBS, The Bethesda System for Reporting Cervical Cytology.

### Cytologic diagnosis and histologic correlation in OTPI

Among 485 Pap cytology cases, 264 (including 151 AGC, 50 HSIL, 50 LSIL, and 13 NILM Pap cases) had histologic follow‐up within 6 months. Of the 151 AGC cases, histologic findings included 53 cases (35.1%) of adenocarcinoma in situ (AIS)/adenocarcinoma (one AIS, nine endocervical adenocarcinomas, 43 endometrial carcinomas) eight cases (5.3%) of CIN2/CIN3/squamous cell carcinoma (SCC; six CIN2/CIN3, two SCCs), 23 cases (15.2%) of CIN1, and 67 (44.4%) benign cases. All NILM Pap cases (13 of 13) had benign histologic follow‐up findings. Fifty cases of HSIL had CIN2+ squamous lesions (43 CIN2/CIN3, seven SCCs), and 50 cases LSIL had CIN1 in the histologic follow‐up. These HSIL and LSIL cases were selected with related biopsy results.

### Cytologic diagnosis and histologic correlation on AICyte‐pathologist findings

Table 5 details the correlation between the AICyte‐pathologist and histologic findings. Among the 143 cases of AGC, 53 (37.1%) were diagnosed as AIS/adenocarcinoma (ADC), 12 (8.4%) were diagnosed as CIN2/CIN3, 20 (14.0%) as were diagnosed as CIN1, and 58 (40.5%) were diagnosed as benign on histologic follow‐up. Among 46 cases of HSIL on AICyte‐pathologist interpretation, all exhibited CIN2/CIN3/SCC on follow‐up. Similarly, all cases of LSIL (51 of 51) by AICyte‐pathologist interpretation had a CIN1 diagnosis in histologic follow‐up. Among 24 cases of NILM on AICyte‐pathologist, 2 (8.3%) cases had CIN1, and all other 22 cases (91.7%) had benign findings on histological follow‐up.

**TABLE 5:** Histologic follow‐up diagnoses correlated with AICyte‐pathologist diagnoses.

TBS categories	No. of FU cases	AIS/ADC, %	CIN2/CIN3/SCC, %	CIN1, %	Benign, %
AGC	143	53 (37.1)	12 (8.4)	20 (14.0)	58 (40.5)
HSIL	46	0 (0.0)	46 (100.0)	0 (0.0)	0 (0.0)
LSIL	51	0 (0.0)	0 (0.0)	51 (100)	0 (0.0)
NILM	24	0 (0.0)	0 (0.0)	2 (8.3)	22 (91.7)
Total	264	53 (20.0)	58 (22.0)	73 (27.7)	80 (30.3)

Abbreviations: ADC, adenocarcinoma (endocervical and endometrial); AGC, atypical glandular cells; AIS, adenocarcinoma in situ; CIN, cervical intraepithelial neoplasia; FU, follow‐up; HSIL, high‐grade squamous intraepithelial lesion; LSIL, low‐grade squamous intraepithelial lesion; NILM, negative for intraepithelial lesion or malignant; No, number; SCC, squamous cell carcinoma; TBS, The Bethesda System for Reporting Cervical Cytology.

### Comparison of the performance characteristics of Pap test interpretations between OTPI and AICyte pathologist

The performance characteristics (sensitivity, specificity, PPV, NPV, accuracy) of the OTPI and AICyte‐pathologist interpretation for detecting CIN2+/AIS/ADC lesions and AIS/ADC lesions are provided in Tables 6 and 7, respectively. In the context of AGC/HSIL Pap tests for detecting CIN2+/AIS/ADC lesions, the OTPI and AICyte‐pathologist interpretation had 100% sensitivity and 100% NPV, whereas the specificity, PPV, and accuracy for detecting CIN2+/AIS/ADC were slightly improved on AICyte‐pathologist interpretation compared with the OTPI (Table 6). If only the performance of detecting glandular lesions was analyzed, the sensitivity and NPV were 100% in both the OTPI and the AICyte‐pathologist interpretation, whereas the specificity, PPV, and accuracy showed better results in the AICyte‐pathologist interpretation than in the OTPI, with increases of 3.80%, 1.96%, and 3.03%, respectively (Table 7).

**TABLE 6:** Diagnostic performance of atypical glandular cells/high‐grade squamous intraepithelial lesion Papanicolaou interpretations for the detection of cervical intraepithelial neoplasia 2 or greater, endocervical adenocarcinoma in situ, and endocervical/endometrial adenocarcinoma lesions.

	Sensitivity, %	Specificity, %	PPV, %	NPV, %	Accuracy, %
OPTI	100.0	41.18	55.22	100.0	65.90
AICyte, final	100.0	49.02	58.73	100.0	70.45

Abbreviations: NPV, negative predictive value; OTPI, original ThinPrep interpretation; PPV, positive predictive value.

**TABLE 7:** Diagnostic performance of atypical glandular cells Papanicolaou interpretations for the detection of endocervical adenocarcinoma in situ and endocervical/endometrial adenocarcinoma lesions.

	Sensitivity, %	Specificity, %	PPV, %	NPV, %	Accuracy, %
OPTI	100.0	53.55	35.10	100.0	62.88
AICyte, final	100.0	57.35	37.06	100.0	65.91

Abbreviations: NPV, negative predictive value; OTPI, original ThinPrep interpretation; PPV, positive predictive value.

## DISCUSSION

As mentioned above, the role of AI in gynecologic cytopathology, as it pertains to the assessment of AGC cases, remains nebulous. This may be a result of multiple factors, including the lack of training data on glandular epithelial lesions; the morphologic diversity of glandular epithelial cells; and variations in smear preparation, staining, and scanning, all of which can lead to difficulties in detecting AGCs by using AI.^13^, ^14^, ^15^, ^16^, ^17^

In the initial stage of our current study, 485 slides were interpreted by a cytopathologist who had 20 years of experience in cytology, using both the microscope and the AICyte system. The interpretation by the same cytopathologist reduced experimental errors caused by other factors, such as differences in interpretation standards. The results indicate show that the Kendall W coefficient reaches 0.845, which means that the TBS classification shows near perfect consistency between the three interpretation methods (OTPI, RTPI, and AICyte; Table 1). In addition, there was strong agreement between the RTPI and the AICyte interpretation of Path‐A (kappa = 0.744). To assess the performance of AGC diagnosis with assisted AICyte among pathologists, two additional pathologists with 10–20 years of working experience reviewed 485 cases on the AICyte system in the second stage of the study. The results produced a Kendall W coefficient of 0.802 among the three pathologists who used AICyte to interpret the slides. This coefficient value signifies strong agreement among the three pathologists. The kappa value of the AICyte‐pathologist and OTPI results was 0.925, with an exact match rate of 95.1%, which specifies strong agreement between the AICyte‐cytopathologist interpretation and the OTPI. This suggests that consistency is maintained between users of the AICyte system and between AICyte and microscopic interpretation.

To once again demonstrate the capability of the AICyte system in terms of efficiency, the time to slide interpretation was re‐evaluated. In the current study, the average time of interpretation was 42.33 seconds. For Path‐A, the reading time decreased when the AICyte system was used compared with a microscope. Because Path‐B and Path‐C used the AICyte system alone, no such distinction can be made; however, their reading times were in the range of those measured for Path‐A. In our initial study using AICyte,^20^ the average time to interpretation was 18.94 seconds per case. Although the current study demonstrated a decreased time to diagnosis with AICyte review compared with microscopic review, the difference was not quite as drastic as that observed in our initial study. We believe the reason for the longer diagnostic time in this study was because of the study design itself, in that most cases selected contained abnormal Pap slides, especially AGC cases. It is expected that cases with atypical findings—those that are not unequivocally benign or malignant—may require increased decision making from the cytopathologist, thus increasing the time to diagnosis. This is in contrast to the initial study, which was enriched in benign/negative cases.^20^ The similar reading time per slide was noted in a recent US study in which 65% ASC‐US and above Pap slides were selected.^19^ This might provide insight into the expected reading time if a pathologist were to receive more atypical cases with the AICyte system, especially if previously proposed models in which a 50% cutoff value were used to remove negative cases from the pathologist workpool.^21^ In either case, the trends show a promising parallel in decreasing reading time compared with the initial AICyte study, which could prompt further study when using the 50% cutoff value. Total reading time would have to be statistically supported and compared with the time and resources required to implement the AICyte system in optimal conditions.^20^ Such factors would include investment in the validation, scanning machines, data storage, and staffing for ongoing quality assurance.

After establishing interpathologist concordance and efficiency, data were reviewed to determine the diagnostic accuracy of AGC and equivalent diagnoses by AICyte. In the study cohort, 53 AIS/ADCs (one AIS, nine endocervical, 43 endometrial) and 58 CIN2+ squamous lesions (49 CIN2/CIN3, nine SCCs) were detected histologically. All AIS/ADC cases had an AGC interpretation on both the original Pap slide and the final AICyte‐pathologist interpretation. Of the 58 cases of CIN2+ squamous lesions, 46 were interpreted as HSIL, and 12 were interpreted as AGC on AICyte. Of 151 AGC cases in the OTPI that had histologic follow up, eight had CIN2+ lesions on histologic follow‐up. These findings indicate the overlapping cytomorphologic features of HSIL and AGC on both AICyte and microscopic examination.

The results of our current study suggest that the specificity and accuracy of the pathologist using the AICyte system were higher than those of the OTPI. Although this may not necessarily contribute to clinical management, its increased specificity compared with the OTPI suggests that it aids in the diagnosis of AGC cytology while providing increased efficiency. The NPV was also higher with the AICyte system; however, the NPV and PPV in this study may not necessarily be representative because the cases selected were not truly randomized from the general population.

While considering the benefits demonstrated from using the AICyte system, it is important to note the limitations of the current study. Although we selected AGC cases and some HSIL, LSIL, and negative cases to approximate a more realistic cohort, ASC‐US, and ASC‐H cases were not included. This may artificially increase the sensitivity and specificity of the study. Another challenge for inclusion was that many cases of cervical and endometrial adenocarcinomas may not have an initial Pap diagnosis of AGCs for cytohistologic correlation. This could result in an overestimation of the sensitivity. One last limitation to note is the rarity of an AGC diagnosis in real‐life practice. In this instance, the smaller sample size affects the sensitivity and specificity compared with the squamous lesion cohorts because of lower statistical power. However, for demonstrating the noninferiority of AICyte interpretation to the OTPI, the data are sufficient and are supportive of real‐world application.

In conclusion, the AICyte system appears to shorten the time to diagnosis compared with manual interpretation. The sensitivity and specificity of detecting AGCs on Pap tests were comparable when using the AI‐assisted system compared with manual microscopy. To our knowledge, the current study represents the largest investigation of AI‐assisted cellular AGC diagnosis to date. This undertaking demonstrates potential/applicability in cervical cytopathology as it pertains to assisting pathologists in recognizing AGCs and equivalent diagnoses in a more efficient manner.

## AUTHOR CONTRIBUTIONS

**Xin Zhang:** Conceptualization; investigation; data curation; methodology; writing—original draft. **Xing Dong:** Conceptualization; data curation; investigation; writing—original draft; methodology. **David Starr:** Conceptualization; writing—original draft; methodology; investigation. **Xinru Bai:** Conceptualization; data curation; investigation; writing—review and editing; methodology. **Yingbin Li:** Conceptualization; investigation; writing—review and editing; data curation; methodology. **Terri E. Jones:** Conceptualization; writing—review and editing; investigation; methodology. **Yixuan Liu:** Conceptualization; writing—review and editing; methodology; investigation. **Xiaoqian Wang:** Conceptualization; writing—review and editing; methodology. **Changzhong Li:** Conceptualization; software; data curation; validation; writing—review and editing. **Xiangchen Wu:** Conceptualization; validation; data curation; software; writing—review and editing. **Xianxu Zeng:** Conceptualization; writing—review and editing; methodology; supervision; investigation; data curation. **Chengquan Zhao:** Conceptualization; investigation; writing—review and editing; supervision; data curation; methodology.

## CONFLICT OF INTEREST STATEMENT

The authors declare no conflicts of interest.
